# Mixed Micellization and Spectroscopic Studies of Anti-Allergic Drug and Non-Ionic Surfactant in the Presence of Ionic Liquid

**DOI:** 10.3390/polym13162756

**Published:** 2021-08-17

**Authors:** Naved Azum, Malik Abdul Rub, Abdullah M. Asiri

**Affiliations:** 1 Center of Excellence for Advanced Materials Research, King Abdulaziz University, Jeddah 21589, Saudi Arabia; malikrub@gmail.com (M.A.R.);; 2 Chemistry Department, Faculty of Science, King Abdulaziz University, Jeddah 21589, Saudi Arabia

**Keywords:** diphenhydramine hydrochloride, TX–45, ionic liquid, mixed micelles, synergism, theoretical models

## Abstract

In drug delivery, surfactants are used to reduce side effects and to increase drug efficiency. The present work aimed to study the interaction of diphenhydramine hydrochloride (anti-allergic drug) with TX–45 (non-ionic surfactant) in the absence and presence of ionic liquid (1-hexyl-3-methylimidazolium chloride). The physicochemical parameters were estimated by the surface tension measurement. Various theoretical models (Clint, Rubingh, Motomura, and Maeda) were applied to determine the attractive behavior between drug and surfactant mixtures at the surface and in bulk. The drug and surfactant mixtures exhibit synergistic behavior in the absence and presence of ionic liquid. Several energetic parameters were also estimated with the assistance of regular solution approximation and pseudo phase separation model that indicate micelle formation and adsorption of surfactant at the surface is thermodynamically advantageous. The morphology of pure and mixture of amphiphiles has been estimated by the Tanford and Israelachvili theories. UV-visible spectroscopy was used to quantify the attractive behavior of the drug with surfactant with the help of a binding constant (*K*).

## 1. Introduction

Drug delivery is the process of administrating drugs to achieve therapeutic effects. From time to time, the researchers try to improve and refine the drug delivery system to increase drug bioavailability, lessen drug deprivation and loss, and thus prevent destructive side effects [1,2,3,4,5]. Numerous drug carriers (surfactants, liposomes, polymers, nanomaterials, and microspheres) have been utilized for drug delivery systems [6,7,8]. Low bioavailability due to the poor solubilization of hydrophobic drugs is the main problem associated with drug therapy. Due to this poor availability, a large quantity of drugs is required to complete the therapy. As a result, accumulation of drug at non-target sites increases the harmful side effects [9]. Surfactants can act as a delivery agent that not only solubilizes the drug but also raises the bioavailability of drug molecules. Surfactants or surface-active agents are amphipathic molecules having polar and non-polar parts in the same body. Due to this dual character, they have one of the exiting features of self-aggregation or micelle formation. The surfactants aggregate into a globular form (micelles) at a specific concentration known as critical micelle concentration (CMC) [10]. Micelles are labile entities formed by the noncovalent association of individual surfactant monomers. Micelles are structurally like lipids having a hydrophilic interface and hydrophobic inner core in a polar solvent. Drugs that cannot normally cross cell membrane can easily pass by being encapsulated in a micelle [11,12,13]. Micelles have several advantages in being used as a drug carrier. (i) They are small in size; (ii) they mostly have low toxicity (iii) they can rest for an extended period in the system (iv) they can solubilize poorly soluble drugs and lessen the harmful side effects of the drugs; (v) they can be arranged in huge quantities effortlessly; and (vi) micelles can form naturally and are energetically steadier regarding association and dissociation. 

The mixture of one or more surfactants is most important to improve self-assembly and their application. In drug delivery, a mixture of surfactants is used to reduce the side effects and to increase the drug efficiency [14]. This enhanced performance of mixed surfactants is due to the interaction among the two or more surfactants. To interpret the effect of mixed surfactants systems on the physicochemical properties of a drug, various research papers have been published [15,16,17,18,19,20,21,22,23]. Earlier, we also investigated the mixed system of numerous amphiphilic drugs with different surfactants or amphiphiles using different techniques [24,25,26,27,28,29,30,31,32,33].

An antihistamine drug (Diphenhydramine hydrochloride, DPH) used in this study can block the proclamation of histamine by histamine-1 receptors and the action of histamine in the body. It is a first-generation anti-allergic drug utilized in cough syrup and tablets [34]. Being first-generation, it has great efficiency in curing allergic problems as compared to a second-generation anti-allergic drug (desloratadine) [35]. Likewise, DPH has anticholinergic, sedative, antiemetic, and antitussive properties. It also acts as a local anesthetic agent because it is an intracellular sodium channel blocker. Like many other surface-active compounds, DPH also shows surface-active properties. The monomers of DPH molecules aggregate to form a micelle but having a low aggregation number [36]. The association of these types of molecules is mainly due to the hydrophobic interaction.

In recent years, a new class of solvents known as ionic liquids (ILs) has gained much attention in the scientific field. The ionic liquid can be defined as the salt in a liquid state that melts at a temperature below 100 °C. These compounds are stable over a wide range of temperatures and have low melting and vapor pressures [37,38,39,40,41]. ILs have been extensively used in the areas of electrochemistry [41], chemical separation [42], polymer chemistry [43], organic synthesis [44], nanomaterials [45], bio-catalysis, and solar cells [46]. ILs having a long alkyl chain show surface activity and behave like a surfactant. ILs, being environmentally benign and amphiphilic, have good scope in pharmaceutical science since they can enhance the permeability of drugs across biological membranes [47]. Based on their physicochemical properties and structural aspects, they are also used as drug reservoirs, excipients and co-solvent.

The main objective of the present work is to investigate the mixed micellization and absorption behavior of antiallergic drug (DPH) in the presence of nonionic surfactant (TX–45). For the future use of such micelles as drug carriers (they are cost-effective and less toxic in comparison to other alternatives), the physicochemical properties of these mixtures are required before their application. Therefore, the present work is an initiative toward amphiphiles-based drug delivery. The pharmaceutical actions of drugs appear to be connected to the drug-membrane interaction. The physicochemical properties of drugs with surfactant micelles can be envisaged as an estimation for their interactions with biological surfaces; thus, amphiphiles micelles have been utilized as mimetics for biomembrane. The literature presents wide understanding of amphiphiles with drugs but there few attempts have been made to date to study the interactions between the drug and amphiphiles in the company of IL. The work on IL has inspired us to discover the effects of IL on the interaction between drug and amphiphiles. In surface science, the novel IL with a 1-alkyl-3-methyl imidazolium cation is used [48,49,50]. This IL has several advantages compared to conventional surfactant; the broadhead group is sturdily striking through π–π interactions and has a solubilizing capacity for a broader range of solutes. Therefore, here, we performed tensiometry and UV-visible spectroscopic measurements to explore the interaction between an anti-allergic drug (DPH) and a non-ionic surfactant (TX–45) in the absence and presence of IL. Various physicochemical parameters were evaluated by using different hypothetical theories. The interaction between the drug and surfactant was also studied by UV-visible spectroscopy.

## 2. Materials and Methods

**Figure 1:**
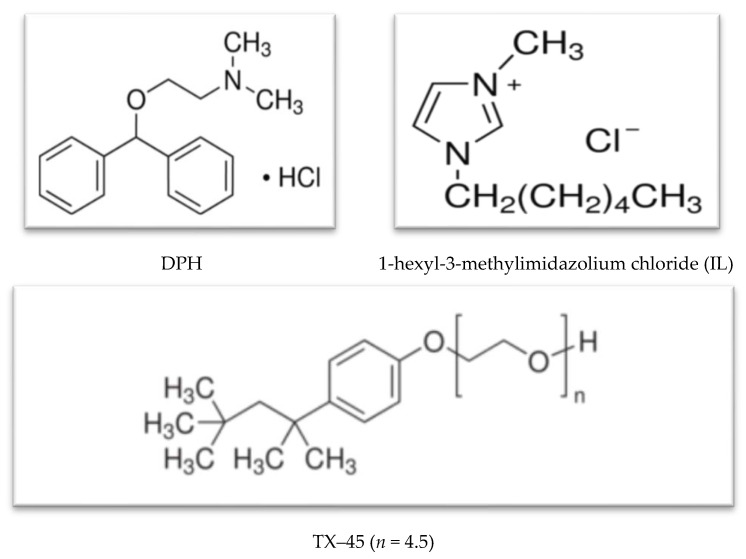
Structure of diphenhydramine hydrochloride (DPH), 1-hexyl-3-methylimidazolium chloride (IL), and triton X–45 (TX–45).

The anti-allergic drug, diphenhydramine hydrochloride, was the product of Molecule-on (New Lynn, Auckland, New Zealand). The non-ionic surfactant, TX–45, and ionic liquid, 1-hexyl-3-methylimidazolium chloride (IL), utilized in this study were purchased from Sigma (St. Louis, MO, USA). The chemicals were utilized as obtained from the supplier, without of additional purification. De-ionized and double distilled water was utilized to prepare all solutions. Specific conductivity was used to check the purity of water (1–6 µS cm^−1^). The pH values were measured by a pH meter (7). The structure of the drug, surfactant, and ionic liquid are given in Figure 1.

### 2.1. Surface Tension Measurements

**Figure 2:**
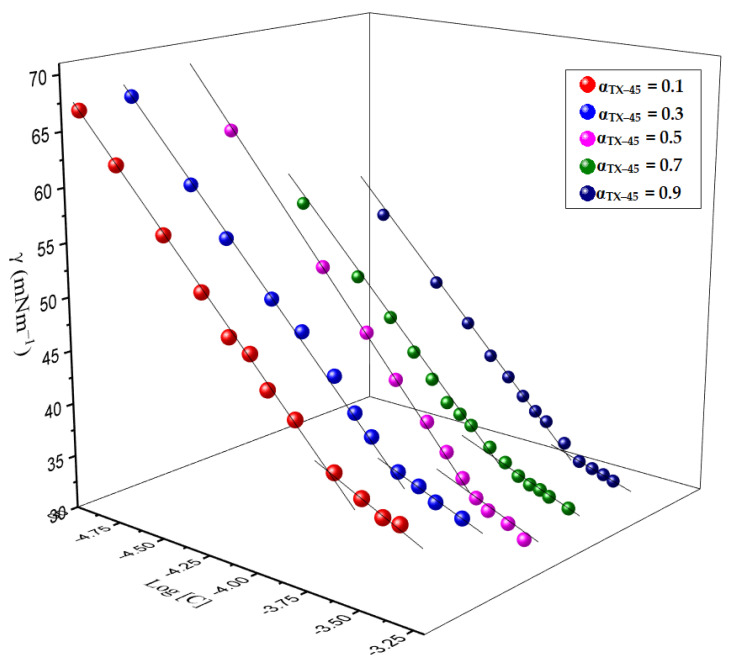
Tensiometric plots of surface tension (*γ*) vs. log amphiphile concentration [*C*] at 298.15.

The surface tension of DPH and the mixture with TX–45 in aqueous solution were recorded by utilizing an attention tensiometer (Sigma 701, Darmstadt, Germany) over the temperature 298.15 *K* (Figure 2). A Citizen CX 220 analytical lab balance was used to weigh compounds for the preparation of the solutions. The instrument was calibrated at regular intervals by ultrapure water. The ring was heated on the ethanol flame until it glowed red before each experiment and, after that, washed with ultra-pure water. In our experiment, we follow the Du Nouy ring method, in which a platinum ring is utilized for the measurement of surface tension.

### 2.2. UV-Visible Spectroscopy

**Figure 3:**
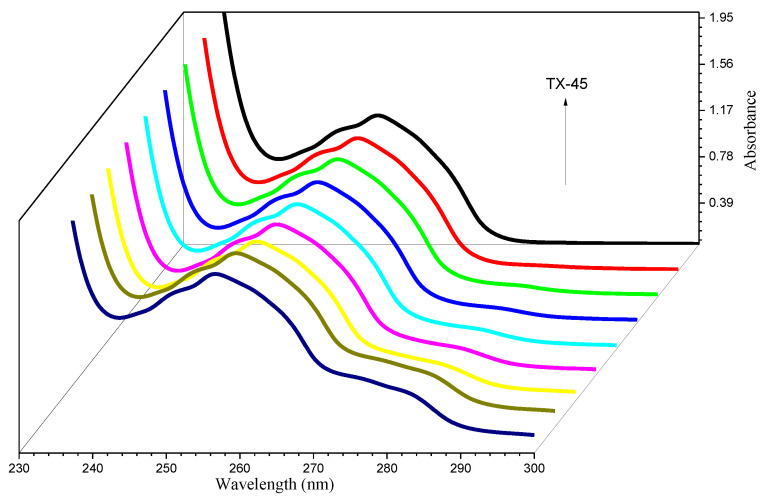
Absorbance spectra of DPH along with rising [TX–45].

A Thermo Scientific, Waltham, MA, USA, Evolution 300 UV-visible spectrophotometer was used to carry out the experiments. All spectra were noted in the spectral range of 230 to 350 nm (Figure 3). The stock solution of DPH (5 mM) was constructed in water. TX–45 solution (5 mM) was prepared in an already prepared drug solution to keep the drug concentration constant throughout. The spectrophotometer was used to analyze these already prepared solutions to observe the effect of TX–45 concentrations on the UV-visible spectra of DPH. To observe the ionic liquid effects, all the solutions were prepared in 25 mM aqueous IL solution instead of water. 

## 3. Results and Discussion

### 3.1. Determination of Critical Micelle Concentration (CMC)

The CMC of pure and mixed systems was computed by plotting the values of surface tension (*γ*) against the concentration (Figure 2). Surface tension is one of the most prominent techniques used to understand the surface and bulk properties of a surface-active compound. The decrease in surface tension after the addition of an amphiphile is due to the displacement of some water molecules by amphiphiles. A plateau region is obtained after the addition of an adequate amount of amphiphiles, which depicts micelle formation. After a critical point, the surface tension values are not constant but slightly decrease due to the existence of impurities. The CMC values of pure and mixed amphiphiles were estimated from the juncture of the two lines manually drawn indicating the high and low concentration regions (Figure 2). The CMC values computed from these curves are given in Table 1. The DPH has higher CMC values than the TX–45, due to the complex structure and ionic nature of DPH. The values of CMC of pure constituents are well-matched with reported values [51,52]. The values of CMC of pure amphiphiles in the presence of 25 mM IL are shown in Table 1. The CMC values of pure amphiphiles (DPH and TX–45) decrease in the presence of 25 mM IL. The IL and DPH have the same charge on the head group, which results in two amphiphiles not interacting appropriately. The counter ions of IL and DPH also lead to decrease in the head group repulsion so that molecules can originate nearer to each other and thus micelle formation can occur at a lower concentration of the drug. Rajput et al., and Pal et al., also described similar behavior [53,54]. However, ion–dipole and hydrogen bonding are responsible for the TX–45 being non-ionic [55].

### 3.2. Clint Theory of Mixed Micellization

The ideal values of CMC (CMC*) were computed by considering the ideal behavior of mixed current systems by Equation (1).

(1) $$\frac{1}{{\mathrm{CMC}}^{*}}=\frac{\alpha_{1}}{{\mathrm{CMC}}_{1}}+\frac{\alpha_{2}}{{\mathrm{CMC}}_{2}}$$

The ideal values along with experimental values at numerous mole fractions of surfactant are listed in Table 1. The nonideal behavior and a constitutional interaction between two amphiphiles are clear from lower values of the experimental CMC than the ideal one, CMC* (Table 1). The experimental CMC values decline with the molar fraction of surfactant (*α*_1_). As the molar fraction of surfactant in binary mixture increases, the electrostatic self-repulsion between the ionic head group of DPH decreases results in a decline in the CMC values of mixed systems. The viewpoint can be supported by the previous investigations [56]. The negative deviation of experimental CMC and ideal CMC in the whole fraction range indicates interaction between the mixed components. In attendance of 25 mM IL, the experimental CMC values were also found to be lower than ideal.

### 3.3. Rubingh Theory of Mixed Micellization

For a non-ideal mixed micellar system, the CMC of a mixture can be calculated by the Rubingh theory.

(2) $$\frac{1}{\mathrm{CMC}}=\frac{\alpha_{1}}{f_{1}{\mathrm{CMC}}_{1}}+\frac{\alpha_{2}}{f_{2}{\mathrm{CMC}}_{2}}$$

The activity coefficients (*f*_1_ and *f*_2_) of amphiphiles can be calculated by the following equations

(3) $$f_{1}=exp\left[\beta {\left(1-X_{1}\right)}^{2}\right]$$

(4) $$f_{2}=exp\left[\beta {\left(X_{1}\right)}^{2}\right]$$

The interaction between two amphiphiles in a mixed micelle can be well understood by analyzing regular solution theory. The micellar mole fraction of amphiphile 1 can be computed by the Rubingh model:

(5) $$\frac{{\left(X_{1}\right)}^{2}\ln\left(\alpha_{1}\mathrm{CMC}/X_{1}{\mathrm{CMC}}_{1}\right)}{{\left(1-X_{1}\right)}^{2}\ln\left[\left(1-\alpha_{1}\right)\mathrm{CMC}/\left(1-X_{1}\right){\mathrm{CMC}}_{2}\right]}=1$$

The values of micellar mole fraction of component 1 ($X_{1}$) are listed in Table 1. On the other hand, the ideal involvement of TX–45 in the DPH + TX–45 mixed micelle can be computed with the help of the micellar mole fraction of surfactant in the ideal state ($X_{1}^{\mathrm{ideal}}$) as follows [57]:

(6) $$X_{1}^{\mathrm{ideal}}=\frac{\alpha_{1}{\mathrm{CMC}}_{2}}{\alpha_{1}{\mathrm{CMC}}_{2}+\alpha_{2}{\mathrm{CMC}}_{1}}$$

The $X_{1}^{\mathrm{ideal}}$ values are given in Table 1. It is clear from the data that the values $X_{1}$ deviate negatively from the ideal values ($X_{1}^{\mathrm{ideal}}$). The higher values of $X_{1}^{\mathrm{ideal}}$ designate that the DPH + TX–45 mixture has a high involvement of drug components than the surfactant. The same results were obtained in the presence of IL. The Rubingh model utilizing the regular solution theory (RST) confirms the attractive or repulsive interaction between two amphiphiles by an interaction parameter *β*, calculated by Equation (7):

(7) $$\beta =\frac{\ln\left(\mathrm{CMC}\alpha_{1}/{\mathrm{CMC}}_{1}X_{1}\right)}{{\left(1-X_{1}\right)}^{2}}$$

The physicochemical interaction between the components before mixing and after mixing at the same condition can be easily understood by the interaction parameter (*β*). When the *β* values are close to zero, means there is no attractive behavior while positive and negative values are a sign of repulsive and attractive behavior of components in a mixed system. According to data in Table 1, values of *β* are negative for the current mixed system at all mole fractions. The non-ionic surfactant (TX–45) has a huge quantity of oxygen atoms wearing unpaired electrons. Therefore, TX–45 molecules tend to interact with positive charge drug molecules coulombically. Table 1 also listed the *β* value in the attendance of IL. In the attendance of IL, *β* is also negative, confirm synergistic behavior. The requirement of synergism for a mixed system is as follows:

(*a*) *β* = negative value (8)

(*b*) *β* > ln (CMC1/CMC2) (9)

It is clear from Table 1 that both the above conditions are followed by our current mixed systems in the nonattendance and attendance of IL. The extent of interaction can also be judged by the most effective parameters and the activity coefficient (Equations (3) and (4)). The computed values of activity coefficients are less than one, showing the nonideality of the current mixed system.

### 3.4. Interfacial Properties of Drug/Surfactant Mixture

When an amphiphile solution is added into the water, the monomers are oriented at the surface and the surface tension of water decreases. The reduction in surface tension is due to the rupturing of hydrogen bonding between water molecules at the surface. If we add more solution, then the further addition gives rise to more reduction in the surface tension values until saturation point. The quantity of amphiphile adsorbed at an interface per unit area can be computed by using Gibbs’ adsorption isotherm. The value of surface excess for the DPH + TX–45 mixture can be calculated by using Equation (10):

(10) $$\Gamma_{max}=-\frac{1}{2.303nRT}\left(\frac{d\gamma }{dlogC}\right)$$

where *C*, *R*, and *T* show the molar concentration, gas constant, and absolute temperature, respectively. *n* stands for the number of constituents at the interface that differ from the surfactant bulk concentration. The values for *n* are taken as three, two and one for the mixture, drug, and TX–45, respectively. $\Gamma_{max}$ is a measure of the effectiveness of the surfactant adsorption at the surface. Higher values of $\Gamma_{max}$ mean extreme stuffing and powerfully squeezing of amphiphiles molecules at the surface. Some surface properties like wetting, foaming, and emulsification are strongly dependent on the effectiveness of adsorption. With the help of $\Gamma_{max}$ values from Equation (10), the minimum area per molecule was also computed:

(11) $$A_{min}=\frac{10^{18}}{N_{A}\Gamma_{max}}\;({\mathrm{nm}}^{2})$$

where *N_A_* is Avogadro’s number. The values of $\Gamma_{max}$ and $A_{min}$ are listed in Table 2.

The $\Gamma_{max}$ value for TX–45 is more than DPH because the long chain has greater surface activity. The $\Gamma_{max}$ values for mixtures are lesser than the values of single amphiphiles. This can be attributed the lowest power of the mixed system to pump the monomers to the surface, which leads to a decline in the concentration of monomers at the surface. The increase in $A_{min}$ for a mixed system suggests a loose mixed monolayer at the surface is formed. However, in the presence of IL, the $\Gamma_{max}$ value slightly decreases (increase in $A_{min}$) for the current mixture. The upsurge or reduction in the values of $\Gamma_{max}$ depend upon numerous aspects, i.e., charge on the head group of the host molecule, hydrogen bonding of IL with the solvent and the occurrence of IL monomers at the interface. The decrease in surface activity of the drug can be understood with the fact that both drug and IL are positively charged, so these molecules of similar charge try to remain separate from each other at the surface monolayer. Thus, the space between monomers at the surface increases ($A_{min}$ increases) and the surface activity decreases [58]. Another reason for the decreasing $\Gamma_{max}$ value after the addition of IL may be the partial adsorption of IL at the surface. IL is also surface-active molecules and have a smaller surface tension value than water. At the interface, some space is occupied by the IL molecules; as a result, the space for drug or other amphiphiles decreases [59]. Adsorption efficiency ($pC_{20})$ is calculated by the equation:

(12) $$pC_{20}=-logC_{20}$$

where *C*_20_ is the efficiency of adsorption of surfactants at the surface. It is the concentration that is mandatory to decrease the surface tension value by 20 mN m^−1^. The values of $pC_{20}$ are listed in Table 2. The current mixed system has larger values of $pC_{20}$, demonstrating that the efficiency of the mixture is greater than the drug. The efficiency of amphiphile was also measured by parameter π_CMC_, computed by Equation (13):

(13) $$\pi_{\mathrm{CMC}}=\gamma_{0}-\gamma_{\mathrm{CMC}}$$

where *γ*_0_ is the surface tension of water, and *γ*_CMC_ is the surface tension at CMC. Higher values of *π*_CMC_ also confirm the earlier explanation. The IL enhances the efficiency of the drug and drug + TX–45 mixed system. Adsorption of IL at the surface offers an additional hydrophobic atmosphere for the drug, raising its propensity for surface adsorption.

### 3.5. Morphology of Drug/Surfactant Mixture

Israelachvili et al. described a relationship for computing the packing parameter (*P*) by using the surface area of amphiphiles [60]:

(14) $$P=\frac{V_{0}}{A_{min}l_{c}}$$

where *l_C_* stands for the extreme operative length of the hydrophobic tail of the monomer, and *V*_0_ is the hydrophobic chain volume computed by using Tanford equations [61]

(15) $$l_{C}=\left(0.154+0.1265(C_{n}-1)\right)\;\mathrm{nm}$$

(16) $$V_{C}=\left(0.0274+0.0269(C_{n-1})\right)\;{\mathrm{nm}}^{3}$$

The values of *P* are listed in Table 3. The measurement of the head group (*A*) is quite difficult, so in our calculations we used $A_{min}$ values instead of *A*. The morphology of a system can be predicted by the value of *P*. For a spherically shaped micelle, the value of *P* should be ≤0.333. The cylindrical (0.333 < *P <* 0.5), vesicle and bilayer (0.5 < *P* < 1) shapes of micelle also exist [60,62,63]. The current TX–45 + DPH mixed system shows that the values for mixed and pure amphiphiles are less than 0.333 (except pure TX–45) in the absence and presence of IL, which confirms spherical micelle formation.

### 3.6. Thermodynamics of the Drug/Surfactant Mixture

The capacity of an amphiphile to form a micelle is reflected in the change in standard Gibbs free energy ($\Delta G_{m}^{o}$). For single and mixed surfactant systems, the standard Gibbs free energy of micellization is given by the following relationship [64].

(17) $$\Delta G_{m}^{o}=RTlnX_{\mathrm{CMC}}$$

The mixing of one amphiphile (TX–45) with the other (DPH) makes the mixed micelle more facile, confirmed by the obtained negative values of $\Delta G_{m}^{o}$ for the current mixed system. On increasing the TX–45 concentration, $\Delta G_{m}^{o}$ values become more negative, specifying the more spontaneous process of mixing. The $\Delta G_{m}^{o}$ values also are calculated by the Maeda model [65]. According to Maeda, the interaction parameter values (*β*) obtained by the Rubingh model incorporated alone into the head group-head group interaction; nonetheless, chain–chain interaction occurs, predominantly when the chains of two or more surfactants are different in length. The CMC of TX–45 in mixture is habitually considerably inferior to the CMC of participated components. This was credited to the decrease in the head group’s repulsion due to the attendance of non-ionic surfactants amid the head group of ionic surfactants. Maeda describes the chain–chain interaction with a parameter *B*_1_, which also describes the stability of the mixture. The free energy of micellization in the presence of ionic surfactant can be obtained by the relation:

(18) $$\Delta G_{M}=RT\left(B_{0}+B_{1}X_{1}+B_{2}X_{1}^{2}\right)$$

where, *B*_0_ = ln CMC_2_, *B*_1_ + *B*_2_ = ln (*C*_1_/*C*_2_) and *B*_2_ = −*β*

The estimated values of all parameters are given in Table 3.

The changes in free energy values calculated by Equation (17) and by Maeda’s Equation (18) agree well. The attained value of $\Delta G_{M}$ of current was found to be negative, considering the spontaneity of the mixed micellation process (Table 3). This result demonstrates the negligible contribution of a degree of counterion binding in the mixed micelle. The drug and TX–45 have different chain lengths; there should be chain-chain interaction stabilizing the micelle. The values of *B*_1_ are higher and negative in the current work, which indicates that the chain–chain interaction plays a foremost character in the stabilization of a micelle. However, the values of *B*_1_ appear to be a function of the composition of a system as well as the head groups.

The values $\Delta G_{m}^{o}$ can be transformed into the standard free energy of adsorption, $\Delta G_{add}^{o}$, at the interface from the relationship (19)

(19) $$\Delta G_{add}^{o}=\Delta G_{m}^{o}-\left(\frac{\pi_{\mathrm{CMC}}}{\Gamma_{max}}\right)$$

here, $\pi_{\mathrm{CMC}}$ is the surface pressure at CMC. The obtained values of $\Delta G_{add}^{o}$ are negative as with $\Delta G_{m}^{o}$ but the magnitude is greater in $\Delta G_{add}^{o}$, revealing that the adsorption is more spontaneous and a primary process while micellization phenomena is a secondary process (Table 3).

The excess free energy of micellization, $\Delta G_{ex}$, can be computed with help of activity coefficients values [66,67,68,69,70]:

(20) $$\Delta G_{ex}=RT\left[X_{1}lnf_{1}+\left(1-X_{1}\right)lnf_{2}\right]$$

The negative values of $\Delta G_{ex}$ suggest the creation of steady mixed micelle rather than micelle formation by the single amphiphiles (Table 3). The negative values also correspond to synergism. The extent of synergism after the mixing of two more amphiphiles can also be evaluated by a thermodynamic parameter, $G_{min}^{s}$ [71]:

(21) $$G_{min}^{s}=A_{min}\gamma_{\mathrm{CMC}}N_{A}$$

The above energetic parameter can be defined as the work required to create a surface area per mole complemented by the changeover from the bulk phase to the surface phase of the solution. The low values of $G_{min}^{s}$ listed in Table 3 suggest a more energetically steady interface is created and extra surface activity is achieved.

### 3.7. UV-Visible Spectroscopy

**Figure 4:**
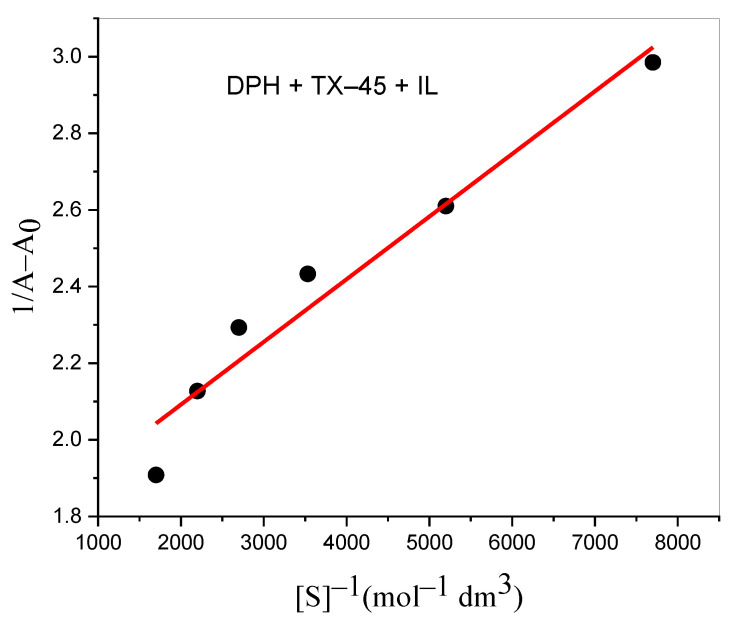
Benesi-Hildebrand graph by utilizing the change in absorption spectra of DPH.

DPH absorption spectra were explored in the attendance of TX–45 to examine the interaction. We also studied the effect of IL on the absorption spectra of DPH + TX–45. Only one characteristic peak at 257 nm was obtained for the DPH (Figure 3). The hyperchromic effect (absorption intensity increases) was observed when we added the TX–45 to the solution of the drug. Thus, the creation of a new complex between DPH and TX–45 was confirmed by the hyperchromic result. The quantitative estimation of the binding can be computed by the Hildebrand Equation (22) [72]:

(22) $$\frac{1}{A-A_{0}}=\frac{1}{K\left(A_{max}-A_{0}\right){\left[S\right]}^{n}}+\frac{1}{A_{max}-A_{0}}$$

where [*S*] is the concentration of TX–45, and *A*, *A*_0_ and *A_max_* are the absorbance values of DPH in the presence of TX–45, in the absence of TX–45, and the absorbance owing to the creation of the drug–surfactant complex. A straight line is obtained on plotting 1/(*A* − *A*_0_) against 1/[*S*] (Figure 4) that also indicates the formation of the 1:1 complex. The binding constant is calculated (intercept/slope) from the Bensei-Hildebrand equation. The obtained binding constant values (*K*) are 2.46 × 10^2^ and 9.38 × 10^3^ M^−1^ for DPH + TX–45 and DPH + TX–45 + IL, respectively. The DPH shows more binding affinity toward TX–45 in the presence of IL, confirmed by the *K* values. It is clear from Figure 4 that the linear plot is obtained when *n* = 1 for DPH + TX–45 mixtures in the presence and absence of IL, indicating 1:1 stoichiometry. The equilibrium processes can be expressed as follows:

$$\mathrm{DPH}+\mathrm{TX}-45\overset{K}{↔}\mathrm{DPH}:\;\mathrm{TX}-45$$

(23) $$K=\frac{\left[\mathrm{DPH}:\mathrm{TX}-45\right]}{\left[\mathrm{DPH}\right]\left[\mathrm{TX}-45\right]}$$

The *K* values are utilized to compute the values of free energy change ($\Delta G_{K}$) by Equation (24)

(24) $$\Delta G_{K}=-RTlnK$$

The values of free energy change are −13.65 and −22.67 kJmol^−1^ for DPH + TX–45 and DPH + TX–45 + IL, respectively. The values of ∆*G_K_* were found to be negative in both cases to confirm the spontaneity of the process of complexation.

## 4. Conclusions

The interaction between DPH and TX–45 is noteworthy in estimation the part of surfactant in biological progressions. Different theoretical models are applied here to know the physicochemical behavior of a drug in the attendance of amphiphile. The experimentally calculated value of CMC is lower than ideally calculated CMC values and the values of CMC decline along with the rise in the mole fraction of surfactant. The interaction parameter values that are negative confirm the synergistic behavior of drug and surfactant mixtures in the nonattendance and attendance of IL. The surface excess ($\Gamma_{max}$) values for mixtures are less than the values of single amphiphiles. That can be attributed lowest power of the mixed system to pump the molecules to the interface, which leads to a decrease in the surface concentration. The $\Delta G_{m}^{o}$ and $\Delta G_{add}^{o}$ values are negative, indicating that the micelle formation and adsorption of surfactants at the surface is thermodynamically advantageous, while the stability of the mixed micelle is validated by the negative value of $\Delta G_{ex}$. For our current mixtures, the values for mixed and single amphiphiles in the attendance and non-attendance of IL are less than 0.333 (except for pure TX–45), confirming spherical micelle formation. The UV-visible spectra of DPH report an enhancement in the absorbance by the addition of surfactant. Electronic absorption spectra in the attendance of IL suggest that, in the presence of IL, the binding of the drug with the surfactant is more efficient, also confirmed by the *K* value.

## Figures and Tables

**Table 1:** Micellar parameters of TX–45 and DPH mixtures in the attendance and non-attendance of 25 mM IL at *T* = 298.15 *K*.

α_1_	CMC (mM)	CMC* (mM)	*X*_1_	$X_{1}^{\mathrm{ideal}}$	−*β*	*f*_1_	*f*_2_	$\ln\left(\frac{{\mathrm{CMC}}_{1}}{{\mathrm{CMC}}_{2}}\right)$
TX–45 + DPH								
0.0	112							−4.89
0.1	0.69	8.39	0.59	0.93	12.45	0.128	0.012	
0.3	0.49	2.94	0.65	0.98	11.41	0.249	0.008	
0.5	0.39	1.79	0.68	0.99	11.36	0.317	0.005	
0.7	0.23	1.28	0.69	0.99	14.09	0.258	0.001	
0.9	0.21	0.99	0.71	0.99	14.72	0.295	0.001	
1.0	0.90							
TX–45 + DPH + 25 mM IL								
0	93.50							−7.71
0.5	0.05	0.08	0.82	0.99	9.66	0.736	0.001	
1.0	0.04							

**Table 2:** Surface parameters of TX–45 and DPH mixtures in the attendance and non-atendance of 25 mM IL at *T* = 298.15 *K*.

α_1_	10^6^ Γ*_max_* (mol m^−2^)	*A_min_* (nm^2^)	*pC*_20_	*γ*_CMC_ (mN m^−1^)	*π*_CMC_ (mN m^−1^)
TX–45 + DPH					
0.0	1.809	0.917	0.940	49.76	20.24
0.1	1.056	1.571	3.324	48.07	21.93
0.3	1.318	1.259	3.367	42.81	27.19
0.5	1.279	1.297	3.961	36.94	33.06
0.7	0.879	1.887	4.428	34.71	35.29
0.9	0.955	1.738	4.591	32.69	37.31
1.0	3.827	0.433	4.910	27.49	42.51
TX–45 + DPH + 25 mM IL					
0	1.256	1.321	0.956	50.27	19.73
0.5	1.315	1.262	5.143	32.23	37.77
1.0	3.249	0.510	5.511	28.31	41.69

**Table 3:** Packing and energetic constraints of TX–45 & DPH mixtures in the attendance and non-attendance of 25 mM IL at *T* = 298.15 *K*.

α_1_	*P*	−*B*_1_	−Δ*G_M_* (kJ mol^−1^)	$-\Delta G_{m}^{o}\;(\mathrm{kJ}\;{\mathrm{mol}}^{-1})$	−Δ*G_ads_* (kJ mol^−1^)	*G_min_* (kJ mol^−1^)	−*G_ex_*
TX–45 + DPH							
0.0	0.23			15.20	26.38	27.49	
0.1	0.13	7.63	29.92	27.99	48.75	45.49	7.44
0.3	0.17	6.59	29.58	28.85	49.47	32.46	6.42
0.5	0.16	6.54	29.63	29.40	55.23	28.85	6.10
0.7	0.11	9.27	31.09	30.71	70.82	39.44	7.46
0.9	0.12	9.90	31.36	30.93	69.99	34.22	7.47
1.0	0.49			27.32	38.43	7.18	
TX–45 + DPH + 25 mM IL							
0	0.16			15.82	31.52	40.00	
0.5	0.17	1.95	35.02	34.44	63.15	24.49	3.50
1.0	0.41			34.92	47.75	8.71	

## Data Availability

Not applicable.
